# Spectroscopic, Spectrometric and Computational Studies of New Lasalocid Derivatives and Their Complexes with Selected Metal Cations

**DOI:** 10.3390/molecules28248085

**Published:** 2023-12-14

**Authors:** Monika Papsdorf, Radosław Pankiewicz

**Affiliations:** Department of Enviromental Physicochemistry, Faculty of Chemistry, Adam Mickiewicz University, Poznań Uniwersytetu Poznańskiego 8, 61-614 Poznań, Poland

**Keywords:** lasalocid, ionophore antibiotics, metal complexes, spectroscopy, theoretical calculations

## Abstract

A series of five esters of lasalocid with neopentyl alcohol (LasNeo), geraniol (LasGeran), 2-ethylhexanol (LasEtHex), eicosanol (LasEico) and vanillyl alcohol (LasVanil) were synthesized and studied by NMR, FT-IR and ESI-MS. Then, their complexes with lithium, sodium and potassium cations were obtained and examined using FT-IR. The analysis of the products confirmed the synthesis of new esters with good yields. The newly obtained compounds, as well as their complexes with monovalent cations, were proved to be stabilized by a strong system of intramolecular hydrogen bonds. The PM6 semiempirical calculations provided information on the heat of formation (HOF) and permitted the making of visual representations of the structures of the newly synthesized esters and their complexes with the investigated cations. All the computational outcomes were consistent with the spectroscopic data.

## 1. Introduction

Polyether antibiotics constitute a very interesting group of substances, showing a broad spectrum of biological activities, including antibacterial, antiprotozoal and antiviral, as well as anti-inflammatory and anticancer ones [1,2,3,4,5,6]. For many years, some of these compounds, such as lasalocid, salinomycin, monensin and semduramycin, have been used in agriculture due to their strong activity against parasites of the genus Eimeria, which causes coccidiosis in birds. Therefore, the introduction of ionophore antibiotics has contributed to better disease control and improved health levels in industrial poultry farming [7].

The molecules of ion carrier ionophores have a non-cyclic structure, but thanks to the presence of a carboxyl group at one end and hydroxyl groups at the other, hydrogen bonds can be formed inside these molecules to form pseudocyclic structures stabilized by these bonds. Ether oxygen atoms are directed towards the interior of the molecule, creating a hydrophilic cavity in which the cation may be complexed [8,9,10,11,12,13]. Certain polyether ionophores are capable of transporting only monovalent cations, e.g., monensin A, while others may also transport divalent cations, e.g., lasalocid acid [14]. In solutions, depending on the solvent and the ion, lasalocid acid can form complexes with different stoichiometries: 1:1, 2:1 and 2:2 [15].

Nowadays, some ionophore antibiotics, like salinomycin, have aroused much interest because of their potent activity against cancer cells, including cancer stem cells [16,17]. For the first time, salinomycin was identified as an effective tumor-targeting agent by Gupta and co-workers [18]. In the following years, salinomycin has been proved to be effective against colon, prostate and gastric cancers and lung adenocarcinoma [19]. Moreover, it has been shown that semisynthetic derivatives of salinomycin have biological activity comparable to that of the unmodified ionophore [20,21,22].

Of note, in 2021, Esben B. Svenningsen and co-workers confirmed the broad-spectrum of antiviral activities of polyether ionophores, including salinomycin, monensin and lasalocid, against the SARS-CoV-2 pandemic [23].

Increasing interest in ionophore antibiotics and the discovery of their new applications have stimulated the search for their new derivatives showing biological activity. These new derivatives should show physicochemical properties at least in one aspect superior to those of the original structure. In this study, our aim was to improve the hydrophobicity of the ionophore molecule. To achieve this, we decided to obtain esters of lasalocid acid with selected alcohols. The newly obtained derivatives were expected to increase its solubility in biological membranes and improve ion transportation, which may be the subject of future research.

## 2. Results and Discussion

### 2.1. ESI Mass Spectrometry

**Figure 1:**
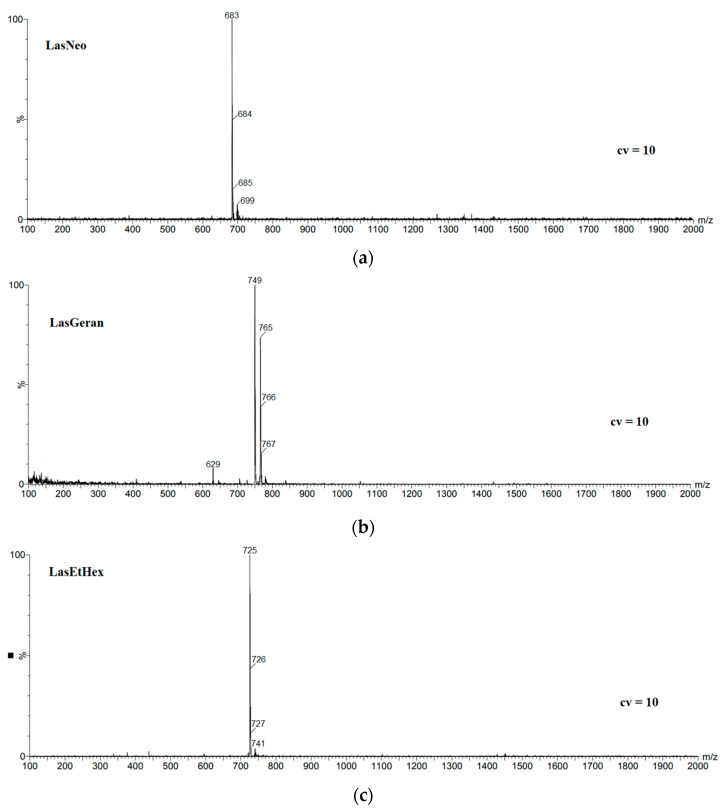

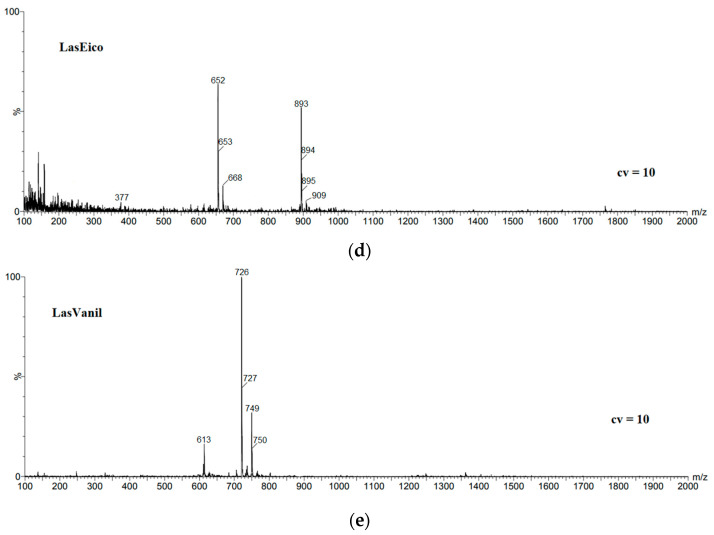
ESI mass spectra of lasalocid esters, (**a**) LasNeo, (**b**) LasGeran, (**c**) LasEtHex, (**d**) LasEico, (**e**) LasVanil.

The mass spectra of LasNeo, LasGeran, LasEtHex, LasEico and LasVanil are shown in Figure 1. The MS spectra were recorded using the technique of electrospray ionization (ESI) at a very low cone voltage (10 V) so that clear molecular peaks corresponding to the formed complexes could be observed (Table 1).

In the spectrum shown in Figure 1a, the most intensive signal at *m*/*z* = 683 is assigned to the LasNeo complex with sodium cations. Accordingly, weaker signals at *m*/*z* = 684 and *m*/*z* = 685 are assigned to isotopic peaks originating from the ester molecule incorporated with ^13^C carbon atoms. Also, very weak signals at *m*/*z* = 699 assigned to the LasNeo complex with the K^+^ cation can be observed.

However, in the mass spectrum of geraniol lasalocid ester (Figure 1b), the strongest signal originating from the LasGeran complex with the sodium cation appears at *m*/*z* = 749, and a slightly weaker signal at *m*/*z* = 765 is assigned to the LasGeran complex with the K^+^ cation, while the weaker signals at *m*/*z* = 766 and *m*/*z* = 767 are assigned to the isotopic peaks coming from the ester molecule incorporated with ^13^C carbon atoms. A very weak signal at *m*/*z* = 629 is attributed to the complex of lasalocid acid with the potassium cation, which is probably a fragment ion.

In the LasEtHex mass spectrum (Figure 1c), the strongest signal at *m*/*z* = 725 was assigned to the LasEtHex complex with the Na^+^ cation. Thus, the weaker signals at *m*/*z* = 726 and *m*/*z* = 727 are the isotopic peaks originating from the ester molecule incorporated with ^13^C carbon atoms. Additionally, a very weak signal visible at *m*/*z* = 741 is assigned to the LasEtHex complex with the potassium cation.

In the mass spectrum of LasEico (Figure 1d), the strongest signal at *m*/*z* = 893 is assigned to the LasEico complex with Na^+^ cations. Therefore, the weaker signals at *m*/*z* = 894 and *m*/*z* = 895 are assigned to an ion containing ^13^C carbon atoms incorporated into the ester molecule. Moreover, a very weak signal appeared at *m*/*z* = 909, assigned to the LasEico complex with the potassium cation. The signal at *m*/*z* = 652 of a similar intensity to that at *m*/*z* = 893 suggests the probable formation of a fragment ion with the Las-C_4_H_9_ + Na^+^ structure. The weaker signal at *m*/*z* = 653 is the isotopic peak originating from the ester molecule incorporated with ^13^C carbon atoms. There is also a very weak signal at *m*/*z* = 668 assigned to the Las-C_4_H_9_ + K^+^ complex. Another very weak signal at *m*/*z* = 377 is consistent with the structure proposed by Lopes et al. [24]. Probably, the ion at *m*/*z* = 377 formed as a result of the internal rearrangement of protons, assisted by sodium.

In the mass spectrum of vanillyl lasalocid ester (Figure 1e), the most intensive signal at *m*/*z* = 726 comes from the LasVanil ester, while the weaker signal at *m*/*z* = 727 is the isotopic peak that originates from the ester molecule incorporated with ^13^C carbon atoms. The low-intensity signal at *m*/*z* = 749 is assigned to the LasVanil complex with the Na^+^ cation, while the weaker signal at *m*/*z* = 750 is also the isotopic peak from an ion containing the ester molecule incorporated with ^13^C carbon atoms. A very weak signal at *m*/*z* = 613 is assigned to the complex of lasalocid acid with the sodium cation, which is probably a fragment ion.

The data obtained through mass spectrometry definitively confirmed the identity of the synthesized chemical compounds. It is important to highlight that the analyzed samples were prepared without incorporating specific sodium and potassium salts. The complex cations that were observed to form likely originated from the glassware used at the synthesis and purification stages. The observation of their formation demonstrates the remarkable capability of the derived lasalocid derivatives to form complexes with monovalent metal cations.

### 2.2. NMR Measurements

**Figure 2:**
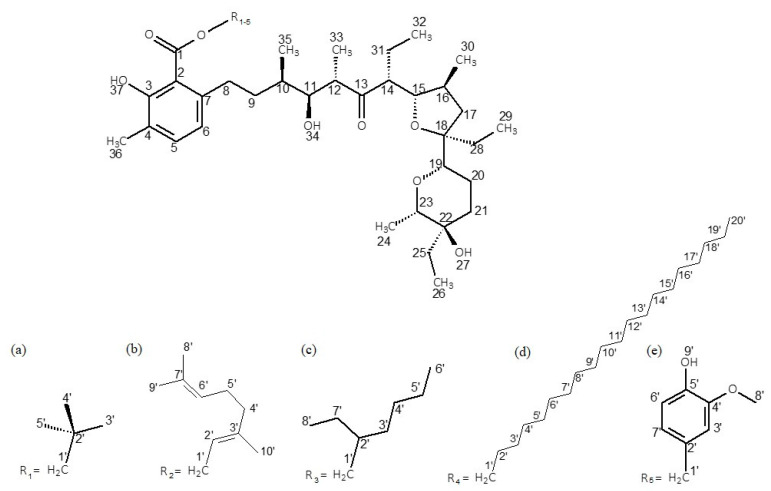
Chemical structures of the studied esters: (**a**) LasNeo, (**b**) LasGeran, (**c**) LasEtHex, (**d**) LasEico, and (**e**) LasVanil.

The Δ^1^H and Δ^13^C NMR data of the esters with neopentyl alcohol, geraniol, 2-ethylenohexanol, eicosanol and vanillyl alcohol in chloroform are collected in Table 2. The numbering of atoms in the molecules is given in Figure 2. The tables with the detailed chemical shifts of lasalocid derivatives are included in Supplementary Materials (Tables S1–S5). Table 2 has been reduced to ensure better readability.

In the ^1^H NMR spectrum, the signals of protons from the OH groups of lasalocid derivatives are found at 11.20, 3.40 and 2.40 ppm. The signal at 11.20 ppm is assigned to the O_37_H proton of the phenolic group, involved in the middle of a strong intramolecular hydrogen bond. The positions of proton signals from the remaining hydroxyl (OH) groups indicate their participation in relatively weak intramolecular hydrogen bonds. In the ^1^H NMR spectrum, the signals from the protons at C_8_ are split, which implies an inhibition of rotation of the salicylic part of the molecule. Additionally, the decoupling of the signals assigned to C_17_H, C_20_H and C_30_H indicates a constriction of the movement of the chain itself. Most probably, this constriction is induced by the formation of a “head-to-tail” hydrogen bond and additional intramolecular hydrogen bonds that stabilize the overall conformation of the molecule. This has been verified through semiempirical and DFT calculations.

The analysis of the ^1^H NMR and ^13^C NMR spectra of LasNeo reveals significant chemical shifts in the C_1′_H signals by 0.50 and 5.59 ppm, which proves the formation of an ester bond with lasalocid, as these are the atoms located directly next to it. We can draw similar conclusions based on the data presented in the tables in Supplementary Materials. The chemical shifts of C_1’_H are clearly visible, and the chemical shifts of C_1’_H in the ^1^H NMR spectra of the other investigated molecules are similar, which proves the formation of the corresponding esters.

In the ^1^H NMR spectrum of LasVanil, we observe a clear shift in the signal assigned to O_37_H from the phenolic group towards stronger fields by 0.39 ppm, which indicates the weakening of this hydrogen bond.

A comparable alteration in the chemical shift is noted in the signal attributed to C_11_H, indicating a modification in the chemical surroundings, likely brought about by a shift in the configuration of the neighboring hydrogen bonds. The signal from C_31_H was also averaged, underscoring the heightened flexibility of the polyether chain. The signals originating from the protons C_9_H, C_17_H and C_20_H are split, similarly to those in the LasH spectrum, indicating that the rotation of the salicylic part is blocked. The locking of rotation can also be deduced from analysis of the other spectra.

### 2.3. FT-IR Measurements

**Figure 3:**
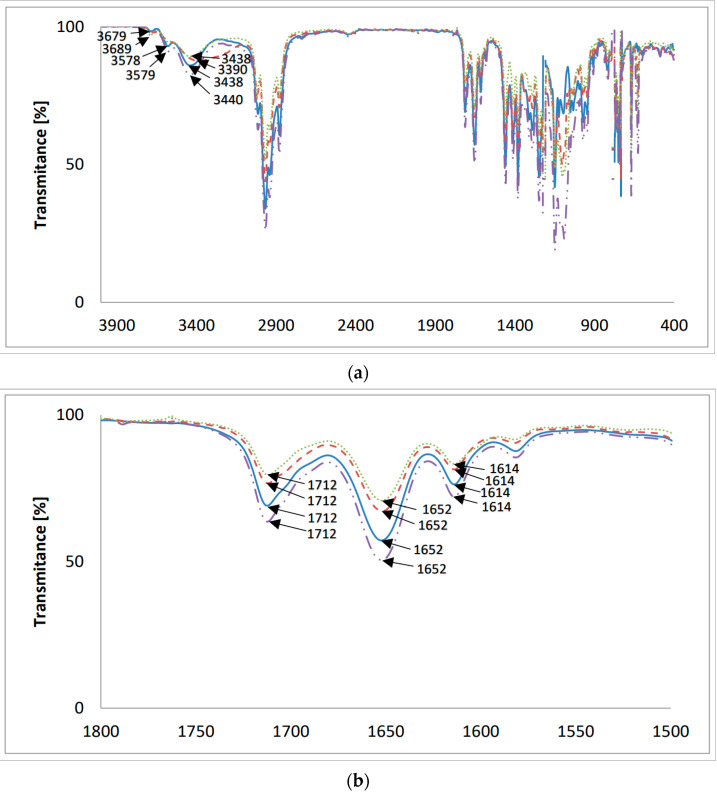
The FT-IR spectra of (**—**) LasNeo and its 1:1 complexes with cations: (**– –**) Li^+^; (⋯) Na^+^; (**-··-**) K^+^; (**a**) 4000–400 cm^−1^; (**b**) 1800–1500 cm^−1^.

**Figure 4:**
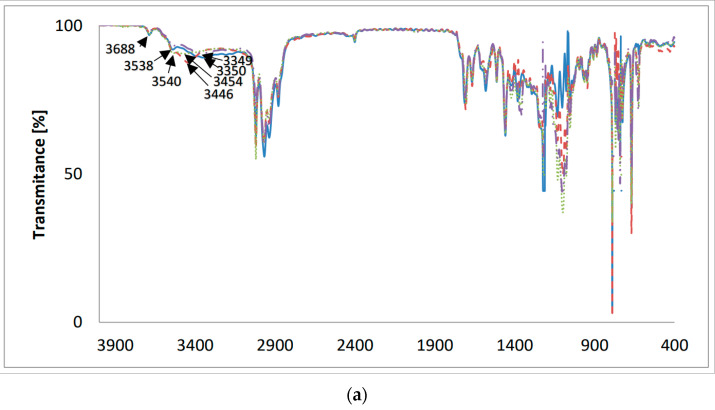

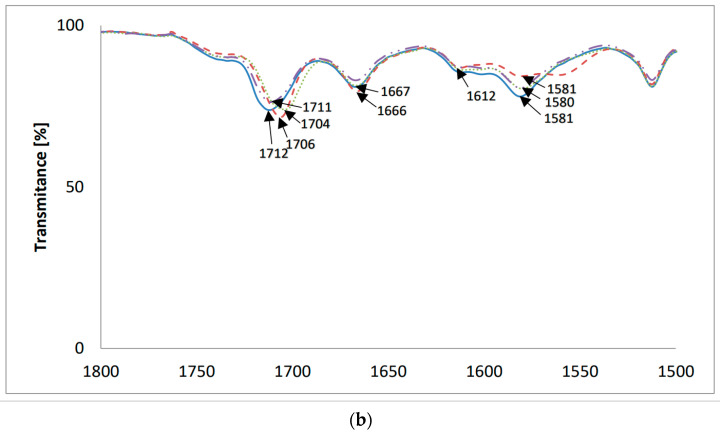
The FT-IR spectra of (**—**) LasVanil and its 1:1 complex with cations: (**– –**) Li^+^; (**⋯**) Na^+^; (**-··-**) K^+^; (**a**) 4000–400 cm^−1^; (**b**) 1800–1500 cm^−1^.

The FT-IR spectra of the new lasalocid esters with neopentyl alcohol (LasNeo) (Figure 3), geraniol (LasGeran) (Supplementary Materials, Figure S6), 2-ethylenohexanol (LasEtHex) (Supplementary Materials, Figure S7), eicosanol (LasEico) (Supplementary Materials, Figure S8) and vanillyl alcohol (LasVanil) (Figure 4) and their 1:1 complexes with monovalent cations in the mid-infrared region are presented in Figure 3 and Figure 4, respectively.

A comparison of the absorption maxima of the individual bands in the FT-IR spectra of lasalocid esters and their 1:1 complexes with the following cations, Li^+^, Na^+^ and K^+^, is presented in Table 3.

The band assigned to the ν(O-H) stretching vibrations of the hydroxyl groups in the spectrum of LasNeo (Figure 3a) appears at 3438 cm^−1^. The analogous bands in the spectra of LasNeo complexes are in the same positions or are slightly shifted to higher wavenumbers, such as 3438 cm^−1^ for LasNeo-Na^+^ and 3440 cm^−1^ for LasNeo-K^+^. The largest shift in the signal coming from an OH group was observed in the spectrum of the LasNeo-Li^+^ complex, moving by 48 cm^−1^ towards the lower wavenumbers, which indicates the strengthening of hydrogen bonds formed by OH groups in these complexes relative to the strength of the hydrogen bonds in the LasNeo ester.

In the FT-IR spectrum of LasNeo (Figure 3b), the absorption maximum of the ν(C=O) carbonyl band is at 1712 cm^−1^, and that of the ν(C=O) carboxyl band is at 1652 cm^−1^. However, for lasalocid complexes with Li^+^, Na^+^ and K^+^ cations, there are no changes in the absorption maximum of the band assigned to the vibrations of the ν(C=O) of the carbonyl group (1712 cm^−1^) or the band ν(C=O) of the carboxyl group (1652 cm^−1^).

For the LasGeran, LasEtHex and LasEico esters and their complexes with the same cations (Supplementary Materials), we have a more or less similar situation, except for the LasEico complex with the lithium cation.

In the FT-IR spectrum of LasVanil (Figure 4), the band assigned to the ν(O-H) stretching vibrations of the hydroxyl groups appears at 3350 cm^−1^, but in the spectra of the LasVanil-Li^+^ and LasVanil-Na^+^ complexes, the bands corresponding to the ν(O-H) stretching vibrations of hydroxyl groups are shifted towards higher wavenumbers, which indicates the weakening of the hydrogen bonds formed by OH groups relative to those in the LasVanil ester. The largest shift in the signal coming from the OH group by 104 cm^−1^ was observed in the spectrum of the LasVanil-Na^+^ complex.

Figure 4b shows the same spectra on an extended scale in the range of 1800–1500 cm^−1^. In the spectrum of LasVanil, the maximum of the ν(C=O) stretching vibration of the ketone group is observed at 1712 cm^−1^, whereas in the spectra of the complexes with lithium or sodium, it is very slightly shifted to 1706 cm^−1^ and 1704 cm^−1^, respectively, indicating no or weak interactions of this ketone group with the cations studied. In the spectrum of the potassium cation complex, a shift in the absorption maximum towards lower wavenumbers only by 1 cm^−1^ was noted.

### 2.4. PM6 and DFT Study

The enthalpies of the formations of LasNeo, LasGeran, LasEtHex, LasEico, LasVanil and the associated species—both complexed and uncomplexed with different monovalent cations—are compiled in Table 4. These findings indicate that the formation of complexes between the investigated esters and cations is thermodynamically advantageous.

A lower ΔHOF value means a higher energy gain is obtained from cation complexation. According to the heat of formation values, the complexation of sodium cation is most preferred, while that of lithium cation is slightly less preferred, which is true for all the compounds studied. The heat of the formation of the complex with the potassium cation is more than half less than that of the complex formation with the sodium cation, which indicates that all esters will preferentially form complexes with Na^+^. This is related to the size of the cavity formed in the ester molecule. Clearly, the ligand structure exhibits a pseudocyclic nature, and the potential size of the cavity is not theoretically constrained. However, intramolecular hydrogen bonds play a crucial role in stabilizing the overall structure.

**Figure 5:**
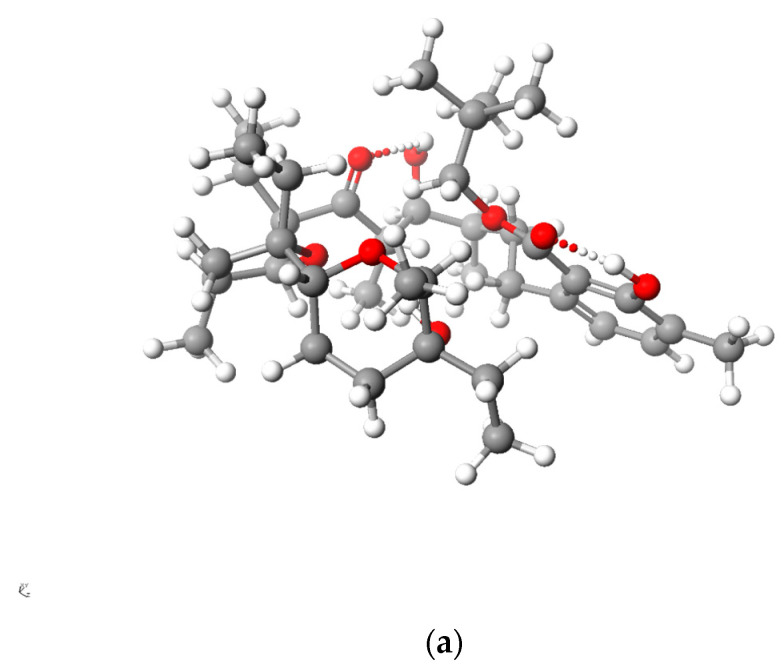

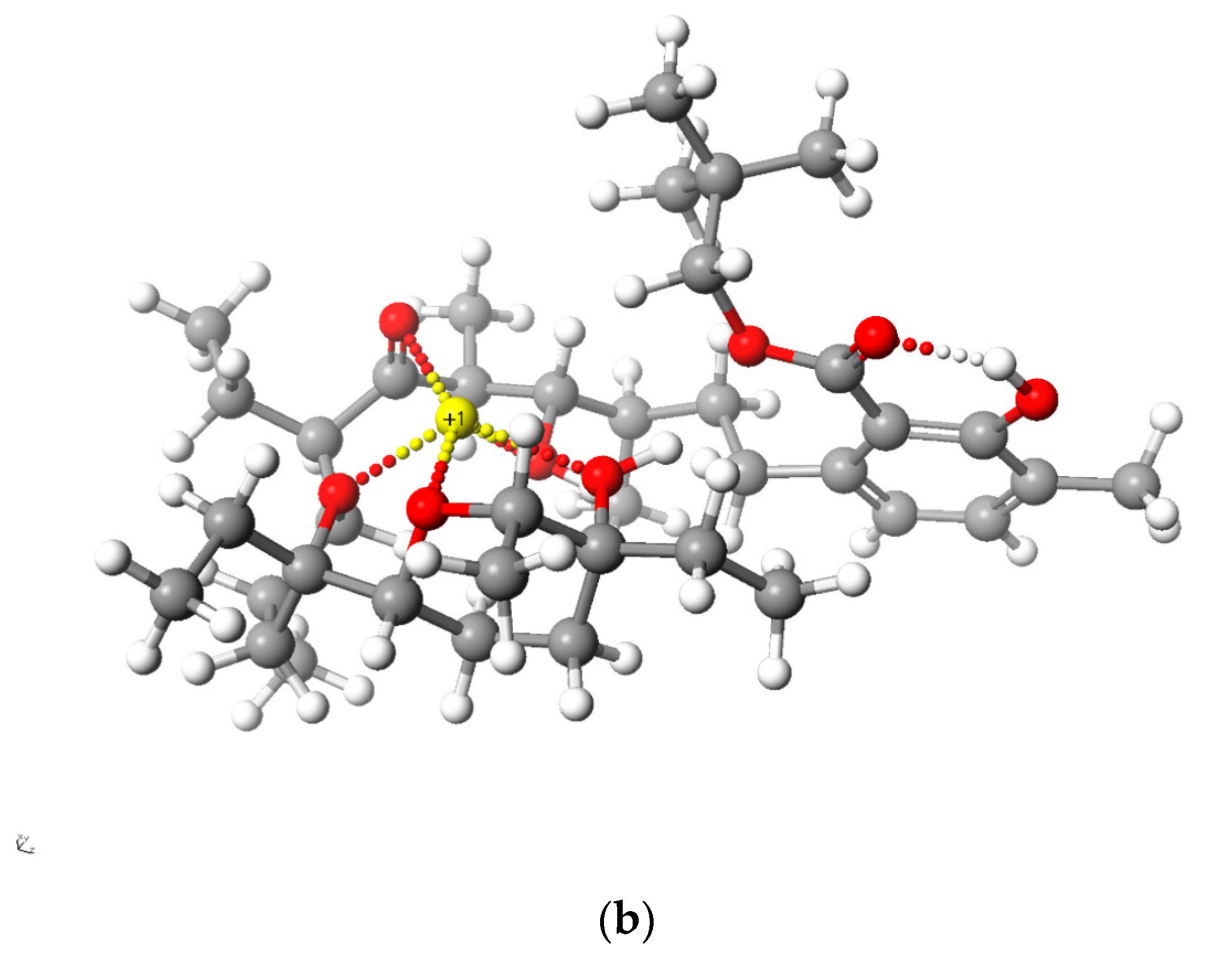
Calculated (DFT) structure of LasNeo (**a**) and its 1:1 complex Li^+^ cation (**b**). Carbon atoms are marked in dark gray, hydrogen in light gray, oxygen in red and lithium in yellow.

**Figure 6:**
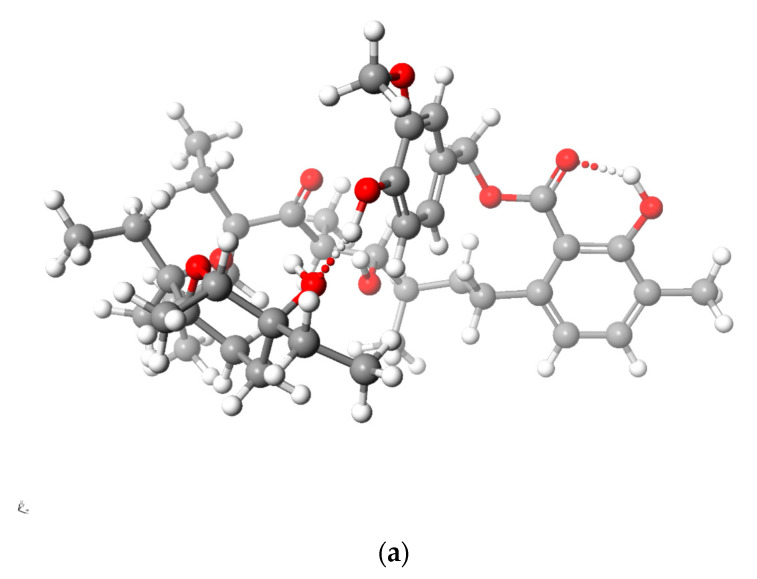

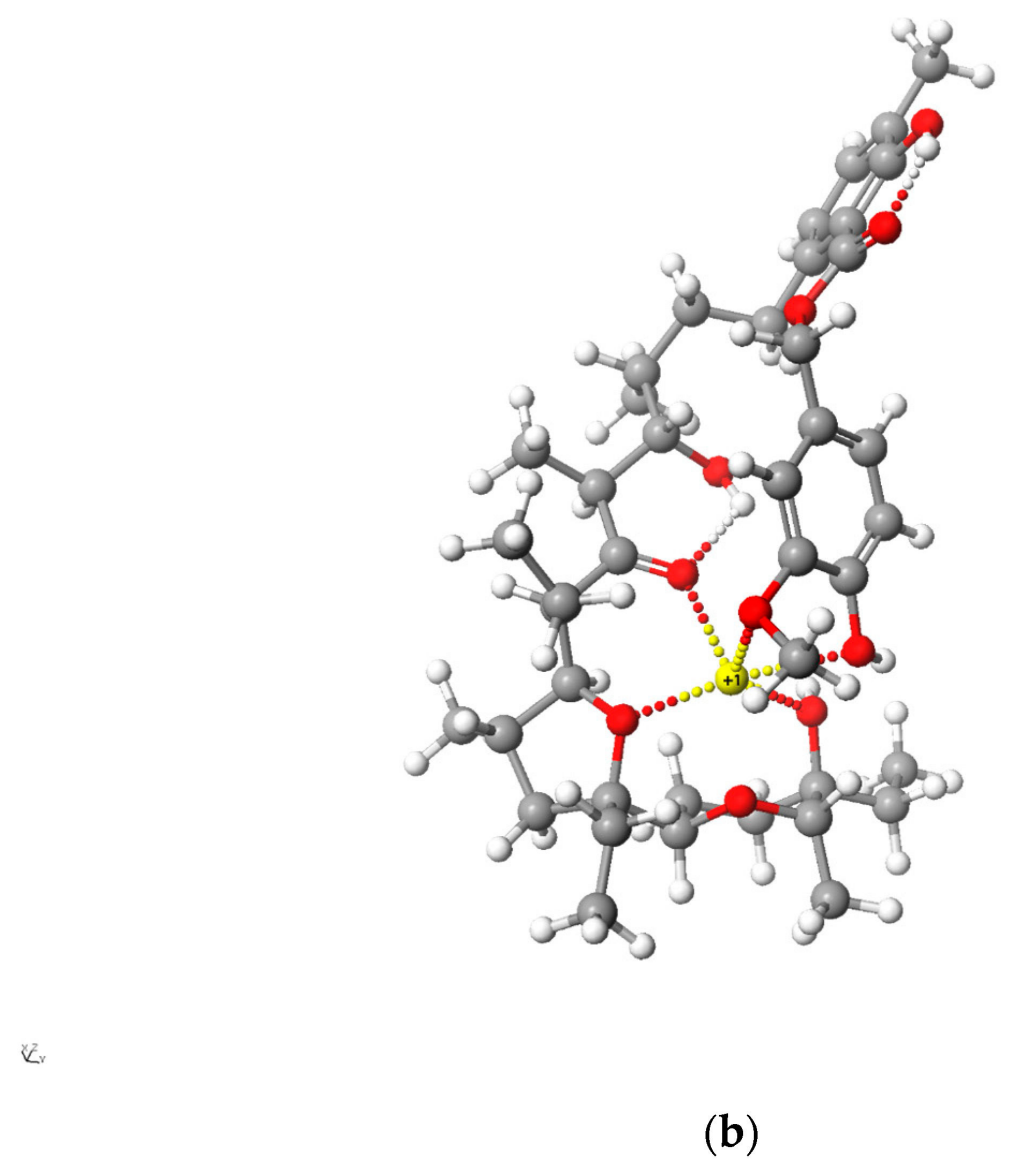
Calculated (DFT) structure of LasVanil (**a**) and its 1:1 complex with Li^+^ cation (**b**). Carbon atoms are marked in dark gray, hydrogen in light gray, oxygen in red and lithium in yellow.

Figure 5 and Figure 6 present the structures of LasNeo and LasNeo–Li^+^ (Figure 5) and LasVanil and LasVanil–Li^+^ (Figure 6) calculated by the DFT methods.

For all the esters, robust intramolecular hydrogen bonds persisted, even following the cation complexation, which is in agreement with the findings from the spectroscopic analysis. However, the configuration of these bonds and the molecular conformation undergo certain alterations, as indicated by the analysis of the ^1^H NMR spectra. For instance, these alterations are evident from the disappearance of the splitting in the signal assigned to the C_31_H proton.

## 3. Materials and Methods

### 3.1. Preparation of Lasalocid Acid

Lasalocid acid was prepared from the animal feed additive (Avatec), which contains lasalocid sodium salts. Preliminarily, 250 g of the feed additive was pre-extracted in 2 L of hexane with Soxhlet apparatus for 12 h to remove dyes and other impurities. Then, relevant extraction in 2 L of methylene chloride with Soxhlet apparatus for the next 12 h was performed to obtain the sodium salt of lasalocid.

Lasalocid acid was obtained from lasalocid sodium salt by extraction with H_2_SO_4_ (pH 1.5) in CH_2_Cl_2_ as described previously [25].

### 3.2. Preparation of the Esters—LasNeo, Las Geran, LasEtHex, LasEico and LasVanil

Lasalocid (0.005 mol) as well as neopentyl alcohol (or geraniol, 2-ethylenohexanol, eicosanol and vanillyl alcohol) (0.005 mol) were dissolved in 50 mL of dichloromethane. The reaction mixture was stirred vigorously for 30 min. Subsequently, 0.005 moles of 1,3-dicyclohexylcarbodiimide (DCC) was introduced to the reaction mixture, which was left stirring overnight at room temperature. The resulting dicyclohexylurea precipitate was separated through filtration, and the remaining solution was concentrated under reduced pressure. The purification process consisted of passing the obtained product through a silica gel column using a CombiFlash NEXGEN 300+ system (Teledyne ISCO) (0 → 30% CH_2_Cl_2_/acetone), which gave us the product as an oil.

After purification, the relevant esters were obtained with the following yields: LasNeo—31.62%; LasGeran—38.46%; LasEtHex—52.76%; LasEico—46.26%; LasVanil—35.22%.

### 3.3. Preparation of Complexes

We have synthesized the relevant complexes with the use of: LiClO_4_, NaClO_4_ and KClO_4_ (Sigma-Aldrich, St. Louis, MI, USA). The solutions were obtained by dissolving the salt and the lasalocid ester in acetonitrile at the ratio 1:1. Acetonitrile was of spectroscopic grade. All the preparations and transfers of solutions were carried out in a carefully dried glovebox.

### 3.4. Elementary Analysis

Elementary analysis was carried out on Vario EL III GmbH equipped with a standard CHN detector. The measurements were repeated triplicate for each ester. For the derivatives: LasNeo–(C_39_H_64_O_8_) (calculated: C 70.80% H 9.77%, found C 70.91% H 9.65%); LasGeran–(C_44_H_70_O_8_) (calculated: C 72.71% H 9.72%, found C 72.78% H 9.81%); LasEtHex–(C_42_H_70_O_8_) (calculated: C 71.70% H 10.06%, found C 71.57% H 10.12%); LasEico–(C_54_H_94_O_8_) (calculated: C 74.50% H 10.92%, found C 74.63% H 11.07%); LasVanil–(C_42_H_62_O_10_) (calculated: C 69.33% H 8.61%, found C 69.11% H 8.40%).

### 3.5. ESI MS Measurements

The electrospray ionization (ESI) mass spectra were recorded with a Waters/Micromass ZQ mass spectrometer. The measurements were performed for the solutions of LasNeo, LasGeran, LasEtHex, LasEico and LasVanil (5 × 10^−4^ mol dm^3^). The samples were prepared in dry acetonitrile and were infused into the ESI source using a Harvard pump at a flow rate of 20 mL min^−1^. Standard ESI mass spectra were recorded at the cone voltages of 10 and 30 V. The source temperature was 120 °C, and the desolvation temperature was 300 °C. Nebulization and desolvation were achieved using nitrogen as the working gas at the flow rates of 100 and 300 dm^3^ h^−1^, respectively. The mass spectra were obtained using the positive ion detection mode, maintaining unit mass resolution with a step size of 1 *m*/*z* unit. The ESI experiments covered a mass range from *m*/*z* = 100 to *m*/*z* = 1300.

### 3.6. NMR Measurements

Nuclear Magnetic Resonance (NMR) spectra were recorded using a BRUKER Avance III HD (Bruker, Billerica, MA, USA) magnetic resonance spectrometer, operating at 400.2 MHz for ^1^H NMR and 100.6 MHz for ^13^C NMR. The ^1^H NMR spectra are presented with chemical shifts relative to Tetramethylsilane (TMS), utilizing the respective residual solvent peaks as the internal standards (CDCl_3_ δ 7.26 ppm). Similarly, the ^13^C NMR spectra are expressed in chemical shifts relative to TMS, with the internal standard being the respective residual solvent peak (CDCl_3_ δ 77.16 ppm). Line broadening parameters were set at 0.5 or 1.0 Hz, and the error in chemical shift values was 0.01 ppm. The assignments of ^1^H and ^13^C NMR signals were accomplished independently for each species on the basis of one- or two-dimensional spectra (COSY, HMQC).

### 3.7. FT-IR Measurement

The infrared spectra in the mid infrared region were recorded in a chloroform solution. The mass of each sample was 10 mg. The FT-IR spectra were obtained using a Bruker IFS 66/s FT-IR spectrophotometer (Bruker, Billerica, MA, USA) equipped with an MCT detector (125 scans, resolution 2 cm^−1^).

### 3.8. Theoretical Calculations

Scigress FJ2.6 (EU 3.1.9) software from Fujitsu, Tokyo, Japan, was employed for the PM6 semiempirical calculations [26]. In all instances, full geometry optimization was conducted without applying symmetry constraints. The DFT calculations were executed using the GAUSSIAN 16 package [27], and the geometries were optimized using Becke’s three-parameter hybrid method with the Lee, Yang and Parr correlation function (B3LYP), along with a 6-311G(d) basis set.

## 4. Conclusions

Lasalocid comprises in its molecular scaffold both the lipophilic as well as the hydrophilic counterparts, which permit the complexation of metal cations.

In order to search for new, alternative lasalocid derivatives showing a superior hydrophobicity, we carried out esterification with the compounds of different structures: neopentyl alcohol, geraniol, 2-ethylenohexanol, eicosanol and vanillyl alcohol. The identity of pure LasNeo, LasGeran, LasEtHex, LasEico and LasVanil ester molecules was confirmed (after purification using flash chromatography). All the new lasalocid derivatives and their complexes maintained the properties of the original ionophore, and they effectively complexed the monovalent cations. On the basis of quantum chemical calculations (PM6), we can conclude that the order of preference for complexing cations is as follows: Na^+^ > Li^+^ > K^+^.

## Figures and Tables

**Table 1:** The main peaks in the ESI mass spectra of the lasalocid complexes at cone voltage 10 V. Signal values assigned to ester complexes with sodium cation are bolded.

Ester	Main Peaks (*m*/*z*)
LasNeo	**683**, 684, 685, 699
LasGeran	629 (w), **749**, 765, 766, 767
LasEtHex	**725**, 726, 227, 741 (w)
LasEico	337 (w), 652, 653, 668 (w), **893**, 894, 909
LasVanil	613 (w), 726, 727, **749**, 750

(w) weak signal.

**Table 2:** Δ^1^H NMR and Δ^13^C NMR chemical shifts (ppm) of LasNeo, LasGeran, LasEtHex, Las Eico and LasVanil in chloroform.

No of Atom	LasNeo		LasGeran		LasEtHex		LasEico		LasVanil	
	Δ^1^H	Δ^13^C	Δ^1^H	Δ^13^C	Δ^1^H	Δ^13^C	Δ^1^H	Δ^13^C	Δ^1^H	Δ^13^C
1	-	0.95	-	0.59	-	1	-	0.81	-	−6.69
2	-	0.26	-	0.05	-	0.13	-	0	-	−0.4
3	-	0.4	-	0.51	-	0.54	-	0.61	-	−9.07
4	-	0.17	-	−0.06	-	0.17	-	0.16	-	0.33
5	0.05	−0.03	0.03	0.02	0.04	0.01	0.04	0.05	0.12	0.99
6	0.1	−0.37	0.06	0.01	0.06	−0.13	0.06	−0.04	0.55	0.61
7	-	−0.22	-	−0.04	-	−0.09	-	0.08	-	−2.11
8	0.09; 0.01	−2.13	−0.09; −0.07	0.64	−0.09; −0.03	0.05	−0.09; −0.07	0.44	−0.09; −0.15	−1.07
9	−0.35	−0.06	−0.39	1.93	−0.31	−0.03	−0.11	−0.04	0.08	−1.23
10	−0.16	−0.32	0	−3.07	−0.16	0.44	0.08	−1	0.2	−2.49
11	0.01	0.04	0.01	0.06	0.01	1.25	0.01	1.25	0.28	0.17
12	0.03	0.66	0.03	0.57	0.03	0.66	0.03	0.6	0.03	0.4
13	-	0.17	-	0.29	-	0.26	-	0.17	-	0.73
14	0.11	−0.1	0.11	−0.09	0.14	−0.09	0.11	−0.11	0.07	0.3
15	0.04	0.73	0.04	0.74	0.04	0.78	0.04	0.76	0	0.49
16	0	0.02	0	−0.54	0	0.15	0.1	0.41	-0.07	−0.29
17	0.14; 0.02	0.32	0.1; 0.29	0.45	0.1; 0.02	0.36	0.1; 0.03	0.34	0.1; 0.02	0.41
18	-	0.34	-	0.4	-	−0.81	-	−0.83	-	0.41
19	0.4	1.51	0.4	1.6	0.4	1.6	0.4	1.66	0.09	2.35
20	0.03; 0.05	0.58	0.04; −0.06	0.58	0.05; 0.06	0.6	0.05; 0.26	0.59	0.04; 0.06	0.47
21	0.07	0.29	0.15	−0.4	0.07	−0.38	0.03	−0.15	0.01	0.08
22	-	0.09	-	0.02	-	0.06	-	0.08	-	−0.02
23	0.05	-	0.05	-	0.05	-	0.05	-	0.05	-
24	0.01	0.22	0.01	−0.1	−0.26	−0.46	0.05	0.26	0.01	0.21
25	−0.22	0.14	0.05	−0.32	0.05	0.14	−0.28	0.15	0.05	0.12
26	−0.84	0.19	−0.09	0.19	0.02	0.2	0.02	0.2	−0.09	0.21
27	−0.17	-	−0.17	-	-0.01	-	−0.1	-	−0.17	-
28	0.2	0.19	0.2	0.77	0.01	−0.05	−0.11	0.41	0.03	0.08
29	0.07	0.05	-0.1	0.03	0.27	0.04	0.15	0.05	0.03	0.08
30	0.06	0.23	0.06	−0.31	0.06	0.09	0.06	0.07	0.02	−0.31
31	1 signal	0.67	1 signal	−0.08	1 signal	0.74	1 signal	0.68	1 signal	0.58
32	0.06	0.37	0.1	0.81	0.02	−0.11	0.02	0.37	−0.02	0.3
33	0.08	0.04	0.04	0.13	0	−0.59	0.04	0.14	−0.04	0.1
34	0.08	-	0.38	-	0.18	-	0.1	-	0.2	-
35	0	0.04	−0.04	0.39	−0.11	−1.12	−0.04	0.06	−0.23	−0.67
36	0.04	0.23	0.04	−0.19	0.04	0.37	0.04	0.36	0.04	0.33
37	0.16	-	0.28	-	0.28	-	0.32	-	0.39	-

**Table 3:** Maxima of the absorption bands observed in the FT-IR spectra of LasNeo, LasGeran, LasEtHex, LasEico, LasVanil and their complexes.

Compounds and Complexes	Maximum Absorption of Vibration Bands:		
	ν(O-H) in the Hydroxyl Group (cm^−1^)	ν(C=O) in the Carbonyl Group (cm^−1^)	ν(C=O) in the Carboxyl Group (cm^−1^)
LasNeo	3438	1712	1652
LasNeo-Li^+^	3390	1712	1652
LasNeo-Na^+^	3438	1712	1652
LasNeo-K^+^	3440	1712	1652
LasGeran	3434	1712	1652
LasGeran-Li^+^	3385	1711	1652
LasGeran-Na^+^	3459	1709	1652
LasGeran-K^+^	3438	1712	1652
LasEtHex	3442	1713	1653
LasEtHex-Li^+^	3406	1711	1653
LasEtHex-Na^+^	3461	1711	1653
LasEtHex-K^+^	3437	1712	1653
LasEico	3433	1712	1652
LasEico-Li^+^	3369	1712	1653
LasEico-Na^+^	3432	1711	1652
LasEico-K^+^	3434	1712	1652
LasVanil	3350	1712	1667
LasVanil-Li^+^	3446	1706	1666
LasVanil-Na^+^	3454	1704	1667
LasVanil-K^+^	3349	1711	1666

**Table 4:** Heat of formation (HOF, kJ/mol) of LasNeo, LasGeran, LasEtHex, LasEico and LasVanil and their complexes with various monovalent cations calculated by PM6 method.

Complex	HOF (kJ/mol)	ΔHOF
LasNeopent	−1901.97	-
LasGeran	−1828.39	
LasEtHex	−1952.06	
LasEico	−2182.47	
LasVanil	−2034.23	
LasNeopent Li^+^ (complexed)	−1620.8	−333.97
LasNeopent Li^+^ (uncomplexed)	−1286.83	
LasNeopent Na^+^ (complexed)	−1711.38	−354.49
LasNeopent Na^+^ (uncomplexed)	−1356.89	
LasNeopent K^+^ (complexed)	−1603.03	−157.43
LasNeopent K^+^ (uncomplexed)	−1445.6	
LasGeran Li^+^ (complexed)	−1561.86	−348.61
LasGeran Li^+^ (uncomplexed)	−1213.25	
LasGeran Na^+^ (complexed)	−1672.57	−389.26
LasGeran Na^+^ (uncomplexed)	−1283.31	
LasGeran K^+^ (complexed)	−1546.81	−174.79
LasGeran K^+^ (uncomplexed)	−1372.02	
LasEtHex Li^+^ (complexed)	−1667.27	−330.35
LasEtHex Li^+^ (uncomplexed)	−1336.92	
LasEtHex Na^+^ (complexed)	−1763.78	−356.8
LasEtHex Na^+^ (uncomplexed)	−1406.98	
LasEtHex K^+^ (complexed)	−1661.97	−166.28
LasEtHex K^+^ (uncomplexed)	−1495.69	
LasEico Li^+^ (complexed)	−1916.75	−349.42
LasEico Li^+^ (uncomplexed)	−1567.33	
LasEico Na^+^ (complexed)	−2015.41	−378.02
LasEico Na^+^ (uncomplexed)	−1637.39	
LasEico K^+^ (complexed)	−1882.66	−156.56
LasEico K^+^ (uncomplexed)	−1726.1	
LasVanil Li^+^ (complexed)	−1758.32	−339.23
LasVanil Li^+^ (uncomplexed)	−1419.09	
LasVanilNa^+^ (complexed)	−1877.76	−388.61
LasVanil Na^+^ (uncomplexed)	−1489.15	
LasVanil K^+^ (complexed)	−1781.33	−203.47
LasVanil K^+^ (uncomplexed)	-1577.86	

## Data Availability

The data is available on request.

## Supplementary Materials

The following are available online at https://www.mdpi.com/article/10.3390/molecules28248085/s1, Figure S1: Structure of neopenthyl lasalocid ester, Figure S2: Structure of geraniol lasalocid ester, Figure S3: Structure of 2-ethylhexanol lasalocid ester, Figure S4: Structure of eicosanol lasalocid ester, Figure S5: Structure of vanillyl lasalocid ester, Figure S6: The FT-IR spectra of (—) LasGeran and its 1:1 complexes with cations: (– –) Li^+^; (⋯) Na^+^; (-··-) K^+^; (a) 4000–400 cm^−1^; (b) 1800–1500 cm^−1^, Figure S7: The FT-IR spectra of (—) LasEtHex and its 1:1 complexes with cations: (– –) Li^+^; (⋯) Na^+^; (-··-) K^+^; (a) 4000–400 cm^−1^; (b) 1800–1500 cm^−1^, Figure S8: The FT-IR spectra of (—) LasEico and its 1:1 complexes with cations: (– –) Li^+^; (⋯) Na^+^; (-··-) K^+^; (a) 4000–400 cm^−1^; (b) 1800–1500 cm^−1^, Table S1: ^1^H NMR and ^13^C NMR chemical shifts (ppm) of LasNeopent in chloroform, Table S2: ^1^H NMR and ^13^C NMR chemical shifts (ppm) of LasGeran in chloroform, Table S3: ^1^H NMR and ^13^C NMR chemical shifts (ppm) of LasEtHex in chloroform, Table S4: ^1^H NMR and ^13^C NMR chemical shifts (ppm) of LasEico in chloroform, Table S5: ^1^H NMR and ^13^C NMR chemical shifts (ppm) of LasVanillyl in chloroform.

## References

[1] Kevin D.A., Meujo D.A.F., Hamann M.T. (2009). Polyether ionophores: Broad spectrum and promising biologically active molecules for the control of drug-resistant bacteria and parasites. Expert Opin. Drug. Discov..

[2] Pankiewicz R., Schroeder G., Przybylski P., Brzezinski B., Bartl F. (2004). Lasalocid polyoxaalkyl esters complexes with Li^+^, Na^+^, K^+^, Rb^+^ and Cs^+^ cations studied by ESI MS and semiempirical methods. J. Mol. Struct..

[3] Huczyński A. (2012). Polyether ionophores—Promising bioactive molecules for cancer therapy. Bioorg. Med. Chem. Lett..

[4] Qi D., Liu Y., Li J., Huang J.H., Hu X., Wu E. (2022). Salinomycin as a potent anticancer stem cell agent: State of the art and future directions. Med. Res. Rev..

[5] Rutkowski J., Brzezinski B. (2013). Structures and properties of naturalny occyrring polyether antibiotics. BioMed. Res. Int..

[6] Schlegel R., Willingham M., Pastan I. (1981). Monensin blocks endocytosis of vesicular stomatitis virus. Bioch. Biophys. Res. Commun..

[7] Russel J.B. (1987). A proposed mechanism of monensin action in inhibiting ruminal bacterial growth: Effects on ion flux and protonmotive force. J. Anim. Sci..

[8] Ferdani R., Gokel G.W., Atwood J.L., Steed J.W. (2004). Ionophores. Encyclopedia of Supramolecular Chemistry.

[9] Fong C.W. (2016). Physiology of ionophore transport of potassium and sodium ions across cell membranes: Valinomycin and 18-crown-6 ether. Int. J. Comput. Biol. Drug Des..

[10] Dorkov P., Pantcheva I.N., Sheldrick W.S., Mayer-Figge H., Petrova R., Mitewa M. (2008). Synthesis, structure and antimicrobial activity of manganese(II) and cobalt(II) complexes of the polyether ionophore antibiotic Sodium Monensin A. J. Inorg. Biochem..

[11] Pankiewicz R., Schroeder G., Brzezinski B., Bartl F. (2004). Spectroscopic and PM5 semiempirical study of New Lasalocid 5-hydroxypentyl Ester and its complexes with monovalent cations. J. Mol. Struct..

[12] Wang Q., Liu N., Deng Y., Guan Y., Xiao H., Nitka T.A., Yang H., Yadav A., Vukovic L., Mathews I.I. (2023). Triepoxide formation by a flavin-dependent monooxygenase in monensin biosynthesis. Nat. Commun..

[13] Pankiewicz R., Schroeder G., Brzezinski B. (2005). Spectroscopic, spectrometric and PM5 semiempirical investigation of new lasalocid 8–hydroxy–3,6–dioxaoctyl ester and its complexes with monovalent cations. J. Mol. Struct..

[14] Westley J.W. (1983). Chemical transformations of polyether antibiotics. Polyether Antibiotics: Naturally Occurring Acid Ionophores.

[15] Schroeder G., Gierczyk B., Brzezinski B., Różalski B., Bartl F., Zundel G., Sośnicki J., Grech E. (2000). ^23^Na NMR and FT-IR studies of sodium complexes with the ionophore lasalocid in solution. J. Mol. Struct..

[16] Fuchs D., Heinold A., Opelz G., Daniel V., Naujokat C. (2009). Salinomycin induces apoptosis and overcomes apoptosis resistance in human cancer cells. Biochem. Biophys. Res. Commun..

[17] Zhang W., Wu J., Li B., Xia J., Wu H., Wang L., Hao J., Zhou Q., Wu S. (2006). Synthesis and biological activity evaluation of 20-*epi*-salinomycin and its 20-*O*-acyl derivatives. RSC Adv..

[18] Gupta P.B., Onder T.T., Jiang G., Tao K., Kuperwasser C., Weinberg R.A., Lander E.S. (2009). Identification of selective inhibitors of cancer stem cells by high-throughput screening. Cell.

[19] Naujokat C., Steinhart R. (2012). Salinomycin as a drug for targeting human cancer stem cells. J. Biomed. Biotechnol.

[20] Versini A., Saier L., Sindikubwabo F., Müller S., Cañeque T., Rodriguez R. (2018). Chemical biology of salinomycin. Tetrahedron.

[21] Antoszczak M. (2019). A comprehensive review of salinomycin derivatives as potent anticancer and anti-CSCs agents. Eur. J. Med. Chem..

[22] Antoszczak M., Huczyński A. (2019). Salimocycin and its derivatives—A new class of multiple-targeted “magic bullets”. Eur. J. Med. Chem..

[23] Svenningsen E.B., Thyrsted J., Blay-Cadanet J., Liu H., Lin S., Moyano-Villameriel J., Olagnier D., Idorn M., Paludan S.R., Holm C.K. (2021). Ionophore antibiotic X-206 is a potent inhibitor of SARS-CoV-2 infection in vitro. Antivir. Res..

[24] Lopes N.P., Gates P.J., Wilkinsc J.P.G., Stauntona J. (2002). Fragmentation studies on lasalocid acid by accurate mass electrospray mass spectrometry. Analyst.

[25] Papsdorf M., Pankiewicz R. (2023). New hydrophilic derivatives of lasalocid and their complexes with selected metal cations. Molecules.

[26] Fujitsu Limited (2013). MO-G Version 1.2A.

[27] Frisch M.J., Trucks G.W., Schlegel H.B., Scuseria G.E., Robb M.A., Cheeseman J.R., Scalmani G., Barone V., Petersson G.A., Nakatsuji H. (2019). Gaussian 16, Revision C.01.

