# Supplementary material for: Spectroscopic, Spectrometric and Computational Studies of New Lasalocid Derivatives and Their Complexes with Selected Metal Cations
Source: Molecules. 2023 Dec 14;28(24):8085. doi: 10.3390/molecules28248085 (PMC10746057; doi:10.3390/molecules28248085)
Supplement: Supplementary file 1 [file molecules-28-08085-s001.zip › molecules-2740123-supplementary.pdf]

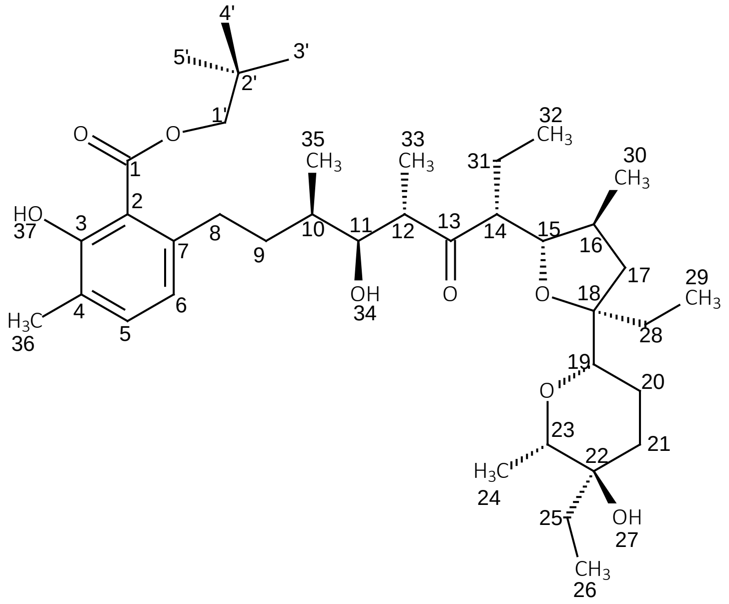

Fig. S1 Structure of neopenthyl lasalocid ester.

Table S1 ^{1}H NMR and ^{13}C NMR chemical shifts (ppm) of LasNeopent in chloroform

No of Atom	LasH		LasNeo			
	^{1}H	^{13}C	^{1}H	^{13}C	◎^{1}H	◎^{13}C
1	–	171.37	–	172.32	–	0.95
2	–	111.54	–	111.80	–	0.26
3	–	160.29	–	160.69	–	0.40
4	–	124.08	–	124.25	–	0.17
5	7.13	134.99	7.18	134.97	0.05	-0.03
6	6.63	121.8	6.73	121.43	0.1	-0.37
7	–	143.43	–	143.21	–	-0.22
8	2.81; 3.00	33.75	2.90; 3.01	31.62	0.09; 0.01	-2.13
9	2.19	35.38	1.84	35.32	-0.35	-0.06
10	1.76	33.95	1.6	33.63	-0.16	-0.32
11	3.47	70.56	3.48	70.60	0.01	0.04
12	2.9	48.99	2.93	49.65	0.03	0.66
13	–	215.28	–	215.45	–	0.17
14	2.71	55.16	2.82	55.06	0.11	-0.10
15	3.79	84.51	3.83	85.24	0.04	0.73
16	~1.6	36.1	1.6	36.12	0	0.02
17	1.50; 1.82	39.22	1.64; 1.84	39.54	0.14; 0.02	0.32
18	–	71.43	–	71.77	–	0.34
19	3.55	72.56	3.95	74.07	0.4	1.51
20	1.45; 1.66	20.64	1.49; 1.71	21.22	0.03; 0.05	0.58
21	1.26	29.86	1.33	30.15	0.07	0.29
22	–	85.8	–	85.89	–	0.09
23	3.74	~77	3.79	75.45	0.05	–
24	1.16	14.02	1.17	14.24	0.01	0.22
25	1.55	30.61	1.33	30.75	-0.22	0.14
26	0.84	6.39		6.58	-0.84	0.19

27	~2.4	–	2.23	–	-0.17	–
28	~1.4	29.3	1.6	29.49	0.2	0.19
29	0.79	8.54	0.86	8.59	0.07	0.05
30	1	15.99	1.06	16.22	0.06	0.23
31	1.40; 1.87	17.91	1.88	18.58	1 signal	0.67
32	0.84	12.53	0.9	12.90	0.06	0.37
33	0.9	13.49	0.98	13.53	0.08	0.04
34	~3.4	–	3.48	–	0.08	–
35	0.9	12.37	0.9	12.41	0	0.04
36	2.19	15.87	2.23	16.10	0.04	0.23
37	11.2	–	11.36	–	0.16	–

No of Atom	Neo		LasNeo			
	^{1}H	^{13}C	^{1}H	^{13}C	◎^{1}H	◎^{13}C
1′	3.287	71.769	3.79	77.36	0.503	5.591
2′	–	32.149	–	26.92	–	-5.229
3′	1.021	24.931	1.06	26.14	–	1.209
4′	1.021	24.931	1.06	26.14	–	1.209
5′	1.021	24.931	1.06	26.14	–	1.209
6′	3.93	–	–	–	–	–

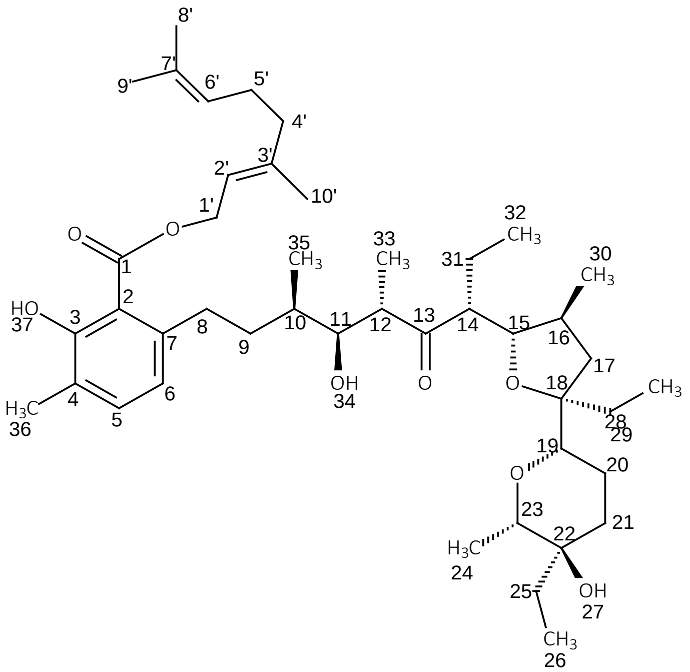

Fig. S2 Structure of geraniol lasalocid ester.

Table S2 ^{1}H NMR and ^{13}C NMR chemical shifts (ppm) of LasGeran in chloroform

No of Atom	LasH		LasGeran			
	^{1}H	^{13}C	^{1}H	^{13}C	◎^{1}H	◎^{13}C
1	–	171.37	–	171.96	–	0.59
2	–	111.54	–	111.59	–	0.05
3	–	160.29	–	160.80	–	0.51

4	–	124.08	–	124.02	–	-0.06
5	7.13	134.99	7.16	135.01	0.03	0.02
6	6.63	121.8	6.69	121.81	0.06	0.01
7	–	143.43	–	143.39	–	-0.04
8	2.81; 3.00	33.75	2.72; 2.93	34.39	-0.09; -0.07	0.64
9	2.19	35.38	1.8	37.31	-0.39	1.93
10	1.76	33.95	1.76	30.88	0	-3.07
11	3.47	70.56	3.48	70.62	0.01	0.06
12	2.9	48.99	2.93	49.56	0.03	0.57
13	–	215.28	–	215.57	–	0.29
14	2.71	55.16	2.82	55.07	0.11	-0.09
15	3.79	84.51	3.83	85.25	0.04	0.74
16	~1.6	36.1	1.6	35.56	0	-0.54
17	1.50; 1.82	39.22	1.60; 2.11	39.67	0.1; 0.29	0.45
18	–	71.43	–	71.83	–	0.40
19	3.55	72.56	3.95	74.16	0.4	1.60
20	1.45; 1.66	20.64	1.49; 1.60	21.22	0.04; -0.06	0.58
21	1.26	29.86	1.41	29.46	0.15	-0.40
22	–	85.8	–	85.82	–	0.02
23	3.74	~77	3.79	77.06	0.05	–
24	1.16	14.02	1.17	13.92	0.01	-0.1
25	1.55	30.61	1.6	30.29	0.05	-0.32
26	0.84	6.39	0.75	6.58	-0.09	0.19
27	~2.4	–	2.23	–	-0.17	–
28	~1.4	29.3	1.6	30.07	0.20	0.77
29	0.79	8.54	0.69	8.57	-0.1	0.03
30	1	15.99	1.06	15.68	0.06	-0.31
31	1.40; 1.87	17.91	1.92	17.83	1 signal	-0.08
32	0.84	12.53	0.94	13.34	0.1	0.81
33	0.9	13.49	0.94	13.62	0.04	0.13
34	~3.4	–	3.78	–	0.38	–
35	0.9	12.37	0.86	12.76	-0.04	0.39
36	2.19	15.87	2.23	15.68	0.04	-0.19
37	11.2	–	11.48	–	0.28	–

No of Atom	Geran		LasGeran			
	^{1}H	^{13}C	^{1}H	^{13}C	◎^{1}H	◎^{13}C
1′	5.41	123.98	5.07	117.89	-0.34	-6.09
2′	4.14	58.84	4.18	59.52	0.04	0.68
3′	–	139.35	–	139.89	–	0.54
4′	2.23	32.09	2.88	36.72	0.65	4.63
5′	1.95	26.68	2.07	26.20	0.12	-0.48
6′	5.09	124.73	4.17	124.18	-0.92	-0.55
7′	–	132.20	–	131.88	–	-0.32
8′	1.68	25.66	1.72	25.62	0.04	-0.04
9′	1.61	17.64	1.64	17.43	0.03	-0.21
10′	1.68	23.42	1.64	25.62	-0.04	2.20
11′	1.57	–	–	–	–	–

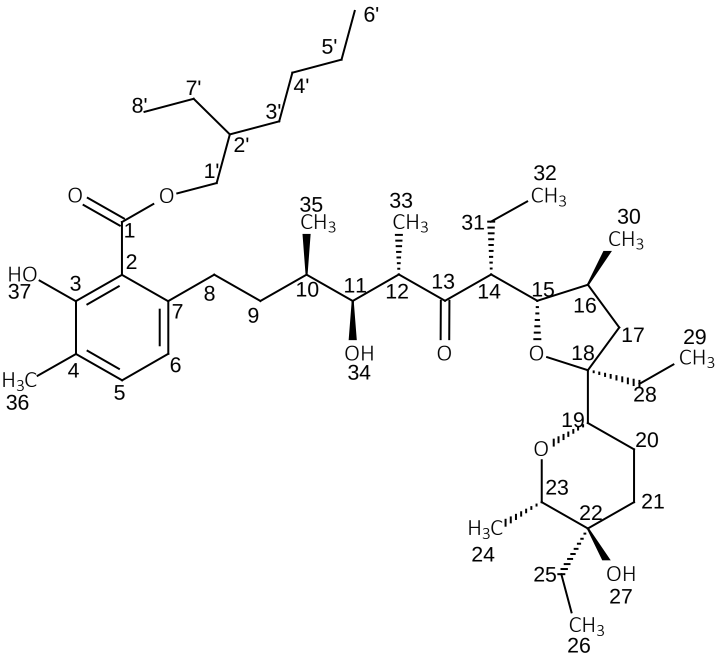

Fig. S3 Structure of 2-ethylhexanol lasalocid ester.

Table S3 ^{1}H NMR and ^{13}C NMR chemical shifts (ppm) of LasEtHex in chloroform

No of Atom	LasH		LasEtHex			
	^{1}H	^{13}C	^{1}H	^{13}C	◎^{1}H	◎^{13}C
1	–	171.37	–	172.37	–	1.00
2	–	111.54	–	111.67	–	0.13
3	–	160.29	–	160.83	–	0.54
4	–	124.08	–	124.25	–	0.17
5	7.13	134.99	7.17	135.00	0.04	0.01
6	6.63	121.8	6.69	121.67	0.06	-0.13
7	–	143.43	–	143.34	–	-0.09
8	2.81; 3.00	33.75	2.72; 2.97	33.80	-0.09; -0.03	0.05
9	2.19	35.38	1.88	35.35	-0.31	-0.03
10	1.76	33.95	1.6	34.39	-0.16	0.44
11	3.47	70.56	3.48	71.81	0.01	1.25
12	2.9	48.99	2.93	49.65	0.03	0.66
13	–	215.28	–	215.54	–	0.26
14	2.71	55.16	2.85	55.07	0.14	-0.09
15	3.79	84.51	3.83	85.29	0.04	0.78
16	~1.6	36.1	1.6	36.25	0	0.15
17	1.50; 1.82	39.22	1.60; 1.84	39.58	0.1; 0.02	0.36
18	–	71.43	–	70.62	–	-0.81
19	3.55	72.56	3.95	74.16	0.4	1.60
20	1.45; 1.66	20.64	1.50; 1.72	21.24	0.05; 0.06	0.60
21	1.26	29.86	1.33	29.48	0.07	-0.38

22	–	85.8	–	85.86	–	0.06
23	3.74	~77	3.79	77.36	0.05	–
24	1.16	14.02	0.9	13.56	-0.26	-0.46
25	1.55	30.61	1.6	30.75	0.05	0.14
26	0.84	6.39	0.86	6.59	0.02	0.20
27	~2.4	–	2.39	–	–0.01	–
28	~1.4	29.3	1.41	29.25	0.01	-0.05
29	0.79	8.54	1.06	8.58	0.27	0.04
30	1	15.99	1.06	16.08	0.06	0.09
31	1.40; 1.87	17.91	1.92	18.65	1 signal	0.74
32	0.84	12.53	0.86	12.42	0.02	-0.11
33	0.9	13.49	0.9	12.90	0	-0.59
34	~3.4	–	3.58	–	0.18	–
35	0.9	12.37	0.79	11.25	-0.11	-1.12
36	2.19	15.87	2.23	16.24	0.04	0.37
37	11.2	–	11.48	–	0.28	–

No of Atom	EtHex		LasEtHex			
	^{1}H	^{13}C	^{1}H	^{13}C	◎^{1}H	◎^{13}C
1′	1.647	42.07	4.34	58.32	2.693	16.25
2′	3.522	65.15	3.56	65.46	0.038	0.31
3′	1.479	30.23	1.84	38.87	0.361	8.64
4′	1.39	29.24	1.33	30.47	-0.06	1.23
5′	1.29	23.19	1.41	23.9	0.12	0.71
6′	0.89	14.12	1.21	14.24	0.32	0.12
7′	1.25	23.43	1.41	23.24	0.16	-0.19
8′	0.89	11.09	0.79	11.01	-0.1	-0.08
9′	2.26	–	–	–	–	–

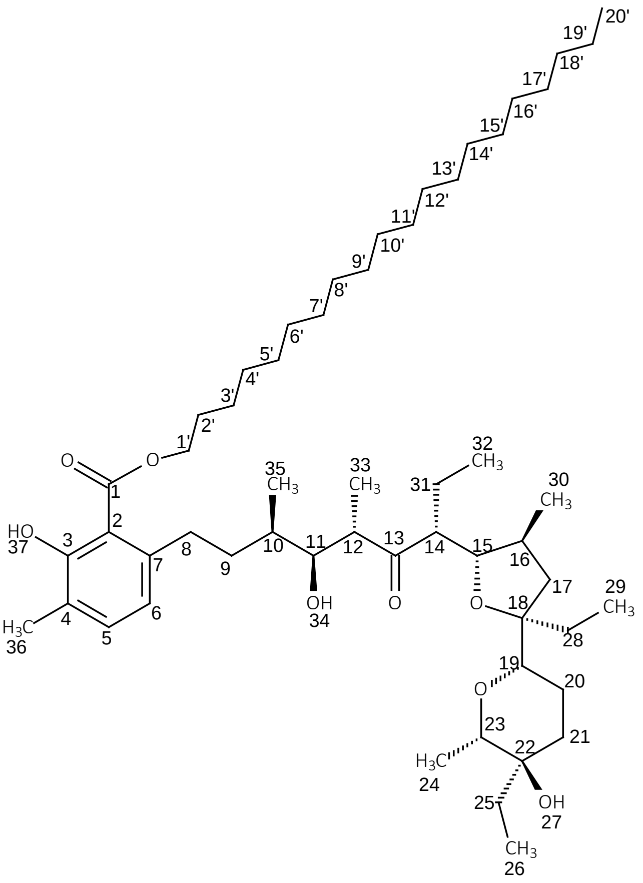

Fig. S4 Structure of eicosanol lasalocid ester.

Table S4 ^{1}H NMR and ^{13}C NMR chemical shifts (ppm) of LasEico in chloroform

No of Atom	LasH		LasEico			
	^{1}H	^{13}C	^{1}H	^{13}C	◎^{1}H	◎^{13}C
1	–	171.37	–	172.18	–	0.81
2	–	111.54	–	111.54	–	0
3	–	160.29	–	160.9	–	0.61
4	–	124.08	–	124.24	–	0.16
5	7.13	134.99	7.17	135.04	0.04	0.05
6	6.63	121.8	6.69	121.76	0.06	-0.04
7	–	143.43	–	143.51	–	0.08
8	2.81; 3.00	33.75	2.72; 2.93	34.19	-0.09; -0.07	0.44
9	2.19	35.38	2.08	35.34	-0.11	-0.04
10	1.76	33.95	1.84	32.95	0.08	-1
11	3.47	70.56	3.48	71.81	0.01	1.25
12	2.9	48.99	2.93	49.59	0.03	0.6
13	–	215.28	–	215.45	–	0.17
14	2.71	55.16	2.82	55.05	0.11	-0.11
15	3.79	84.51	3.83	85.27	0.04	0.76
16	~1.6	36.1	1.7	36.51	0.1	0.41
17	1.50; 1.82	39.22	1.60; 1.85	39.56	0.1; 0.03	0.34
18	–	71.43	–	70.6	–	-0.83

19	3.55	72.56	3.95	74.22	0.4	1.66
20	1.45; 1.66	20.64	1.50; 1.92	21.23	0.05; 0.26	0.59
21	1.26	29.86	1.29	29.71	0.03	-0.15
22	–	85.8	–	85.88	–	0.08
23	3.74	~77	3.79	77.06	0.05	–
24	1.16	14.02	1.21	14.28	0.05	0.26
25	1.55	30.61	1.27	30.76	-0.28	0.15
26	0.84	6.39	0.86	6.59	0.02	0.2
27	~2.4	–	2.3	–	-0.1	–
28	~1.4	29.3	1.29	29.71	-0.11	0.41
29	0.79	8.54	0.94	8.59	0.15	0.05
30	1	15.99	1.06	16.06	0.06	0.07
31	1.40; 1.87	17.91	1.88	18.59	1 signal	0.68
32	0.84	12.53	0.86	12.9	0.02	0.37
33	0.9	13.49	0.94	13.63	0.04	0.14
34	~3.4	–	3.5	–	0.1	–
35	0.9	12.37	0.86	12.43	-0.04	0.06
36	2.19	15.87	2.23	16.23	0.04	0.36
37	11.2	–	11.52	–	0.32	–

No of Atom	Eico		LasEico			
	^{1}H	^{13}C	^{1}H	^{13}C	◎^{1}H	◎^{13}C
1′	3.63	63.11	4.36	66	0.73	2.89
2′	1.56	32.89	1.27	32.07	-0.29	-0.82
3′	1.26	25.82	1.29	25.88	0.03	0.06
4′	1.26	29.51	1.29	29.52	0.03	0.01
5′	1.26	29.76	1.29	29.71	0.03	-0.05
6′	1.26	29.76	1.29	29.71	0.03	-0.05
7′	1.26	29.76	1.29	29.71	0.03	-0.05
8′	1.26	29.76	1.29	29.71	0.03	-0.05
9′	1.26	29.76	1.29	29.71	0.03	-0.05
10′	1.26	29.76	1.29	29.71	0.03	-0.05
11′	1.26	29.76	1.29	29.71	0.03	-0.05
12′	1.26	29.76	1.29	29.71	0.03	-0.05
13′	1.26	29.76	1.29	29.71	0.03	-0.05
14′	1.26	29.76	1.29	29.71	0.03	-0.05
15′	1.26	29.76	1.29	29.71	0.03	-0.05
16′	1.26	29.76	1.29	29.71	0.03	-0.05
17′	1.26	29.43	1.29	29.44	0.03	0.01
18′	1.26	31.98	1.29	30.12	0.03	-1.86
19′	1.26	22.74	1.29	22.84	0.03	0.1
20′	0.89	14.13	0.89	14.25	0	0.12
21′	1.41	–	–	–	–	–

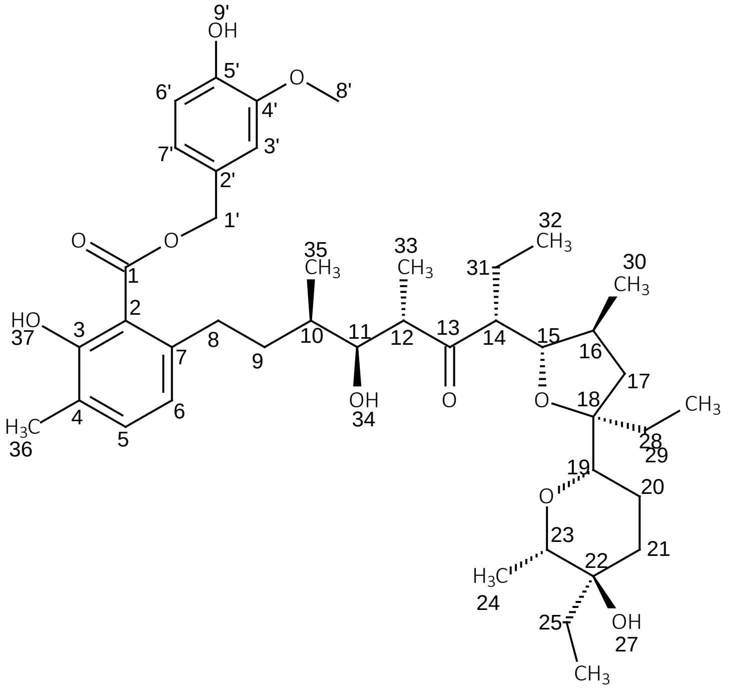

Fig. S5 Structure of vanillyl lasalocid ester.

Table S5 ^{1}H NMR and ^{13}C NMR chemical shifts (ppm) of LasVanillyl in chloroform

No of Atom	LasH		LasVanillyl			
	^{1}H	^{13}C	^{1}H	^{13}C	◎^{1}H	◎^{13}C
1	–	171.37	–	164.68	–	-6.69
2	–	111.54	–	111.14	–	-0.40
3	–	160.29	–	151.22	–	-9.07
4	–	124.08	–	124.41	–	0.33
5	7.13	134.99	7.25	135.98	0.12	0.99
6	6.63	121.8	7.18	122.41	0.55	0.61
7	–	143.43	–	141.32	–	-2.11
8	2.81; 3.00	33.75	2.72; 2.85	32.68	-0.09; -0.15	-1.07
9	2.19	35.38	2.27	34.15	0.08	-1.23
10	1.76	33.95	1.96	31.46	0.2	-2.49
11	3.47	70.56	3.75	70.73	0.28	0.17
12	2.9	48.99	2.93	49.39	0.03	0.40
13	–	215.28	–	216.01	–	0.73
14	2.71	55.16	2.78	55.46	0.07	0.30
15	3.79	84.51	3.79	85.00	0	0.49
16	~1.6	36.1	1.53	35.81	–0.07	-0.29
17	1.50; 1.82	39.22	1.60; 1.84	39.63	0.1; 0.02	0.41
18	–	71.43	–	71.84	–	0.41
19	3.55	72.56	3.64	74.91	0.09	2.35
20	1.45; 1.66	20.64	1.49. 1.72	21.11	0.04; 0.06	0.47
21	1.26	29.86	1.27	29.94	0.01	0.08
22	–	85.8	–	85.78	–	-0.02

23	3.74	~77	3.79	77.00	0.05	–
24	1.16	14.02	1.17	14.23	0.01	0.21
25	1.55	30.61	1.6	30.73	0.05	0.12
26	0.84	6.39	0.75	6.60	-0.09	0.21
27	~2.4	–	2.23	–	-0.17	–
28	~1.4	29.3	1.43	29.38	0.03	0.08
29	0.79	8.54	0.82	8.62	0.03	0.08
30	1	15.99	1.02	15.68	0.02	-0.31
31	1.40; 1.87	17.91	1.88	18.49	1 signal	0.58
32	0.84	12.53	0.82	12.83	-0.02	0.30
33	0.9	13.49	0.86	13.59	-0.04	0.10
34	~3.4	–	3.6	–	0.20	–
35	0.9	12.37	0.67	11.70	-0.23	-0.67
36	2.19	15.87	2.23	16.20	0.04	0.33
37	11.2	–	11.59	–	0.39	–

No of Atom	Vanillyl		LasVanillyl			
	^{1}H	^{13}C	^{1}H	^{13}C	◎^{1}H	◎^{13}C
1′	6.717	114.32	7.26	126.25	0.543	11.93
2′	–	132.99	–	133.04	–	0.05
3′	6.885	109.99	6.79	110.55	-0.095	0.56
4′	–	146.71	–	146.17	–	-0.54
5′	–	145.32	–	144.49	–	-0.83
6′	4.382	65.27	4.65	65.09	0.268	-0.18
7′	6.708	120.25	6.84	119.15	0.132	-1.1
8′	3.751	55.94	3.6	53.88	-0.151	-2.06
9′	8.79	–	–	–	–	–
10′	5.02	–	–	–	–	–

a)

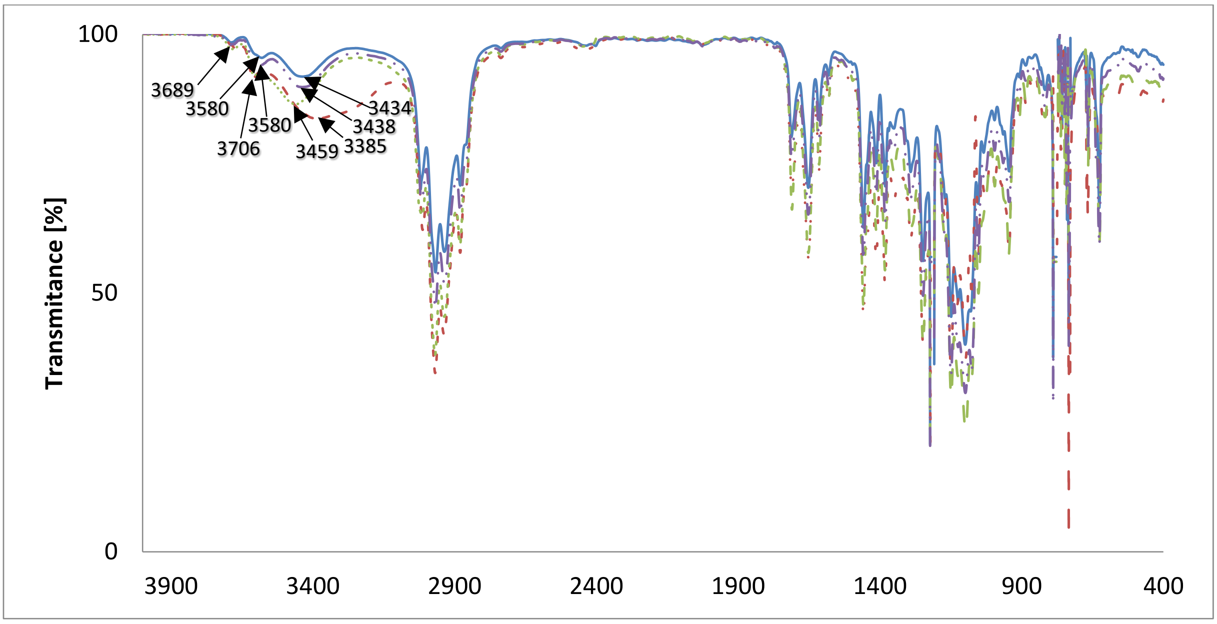

b)

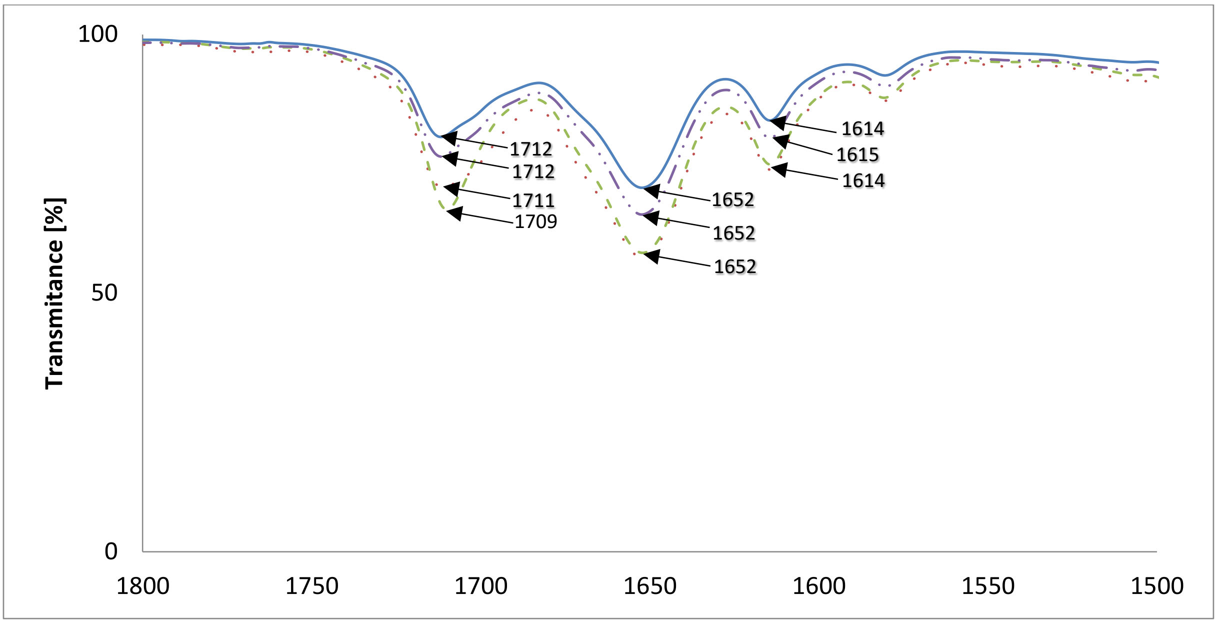

Fig. S6 The FT-IR spectra of (—) LasGeran and its 1:1 complexes with cations: (– –) Li^{+}; (···) Na^{+}; (-··-) K^{+}; (a) 4000–400 cm^{-1}; (b) 1800–1500 cm^{-1}

a)

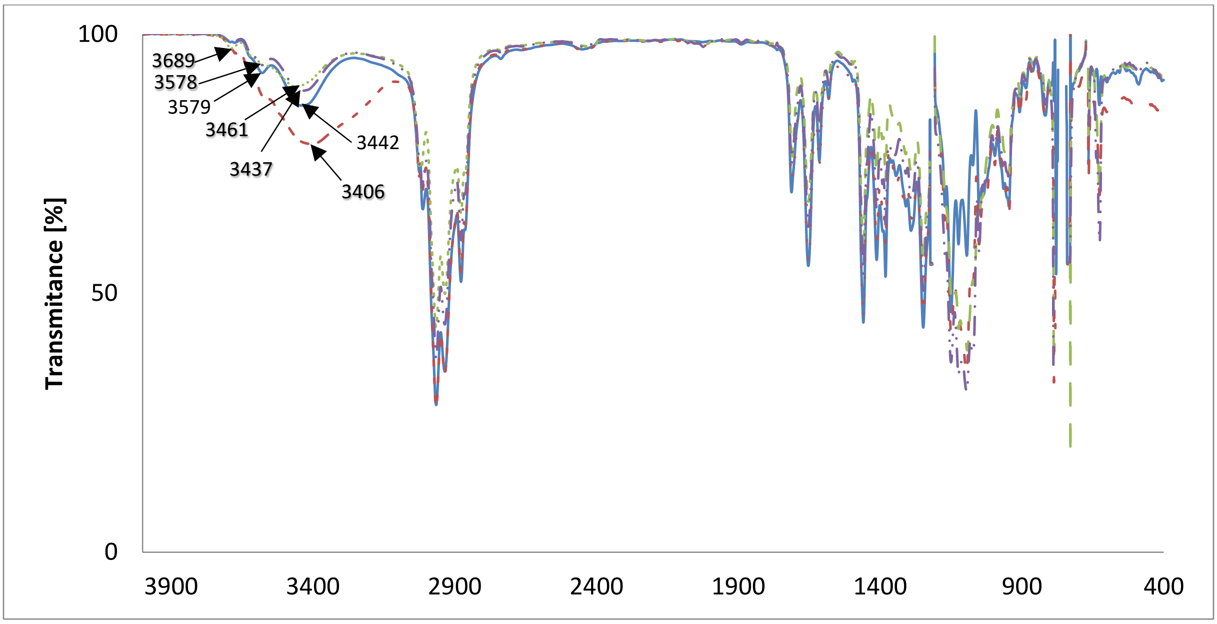

b)

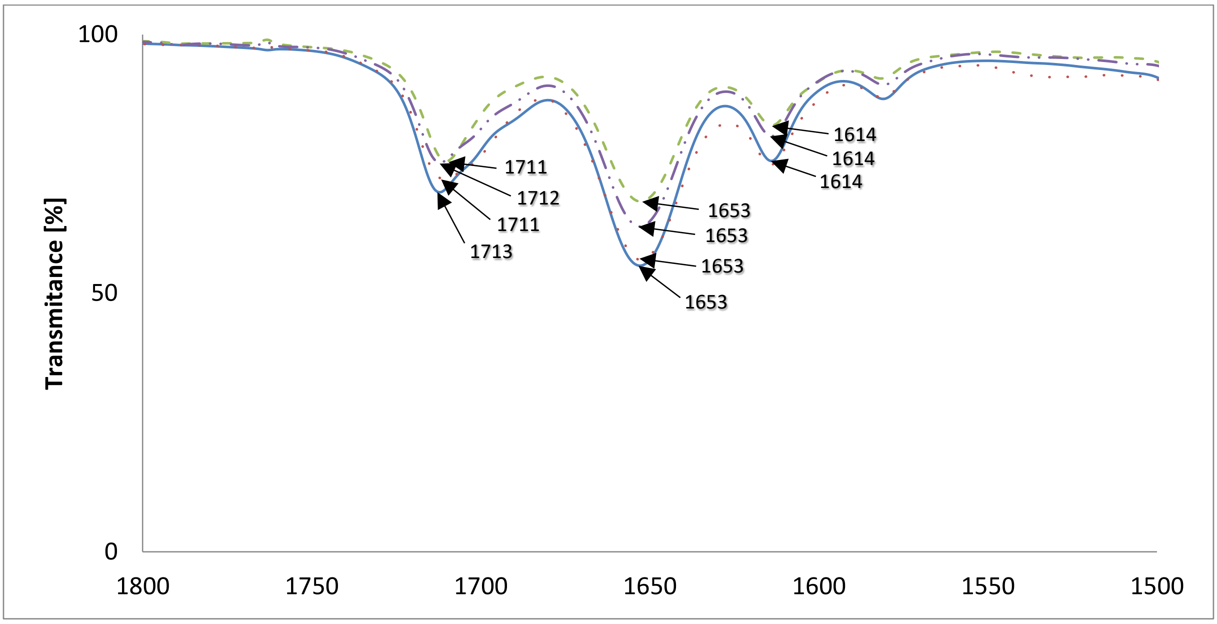

Fig. S7 The FT-IR spectra of (—) LasEtHex and its 1:1 complexes with cations: (– –) Li^+; (···) Na^+; (-··-) K^+; (a) 4000–400 cm^{-1}; (b) 1800–1500 cm^{-1}

a)

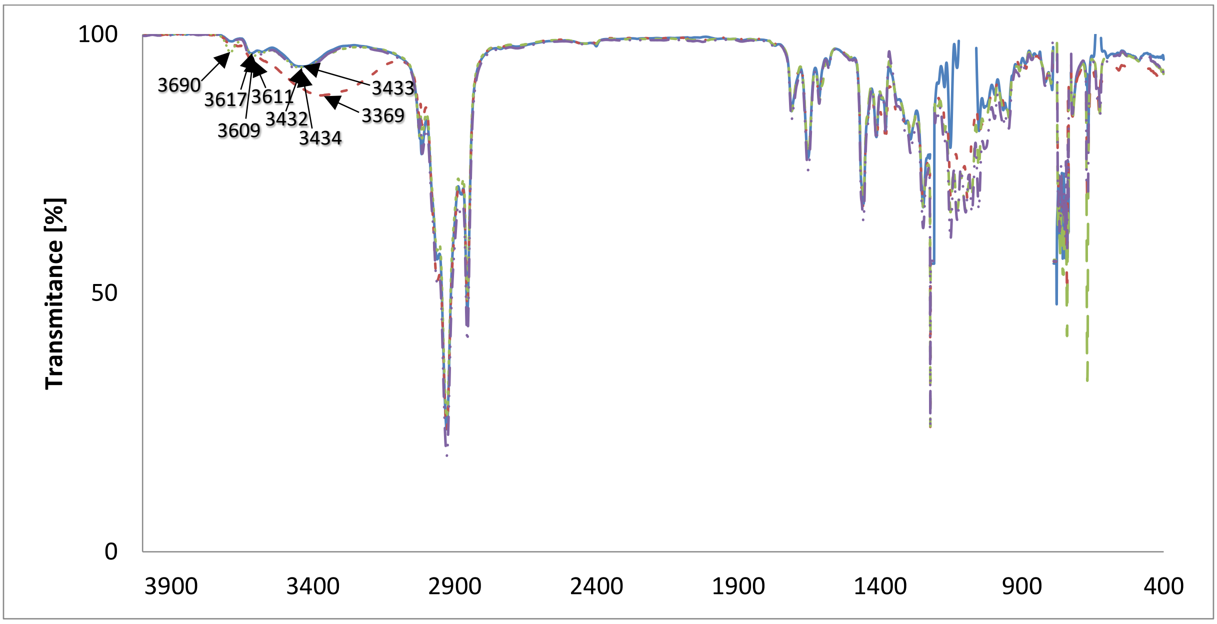

b)

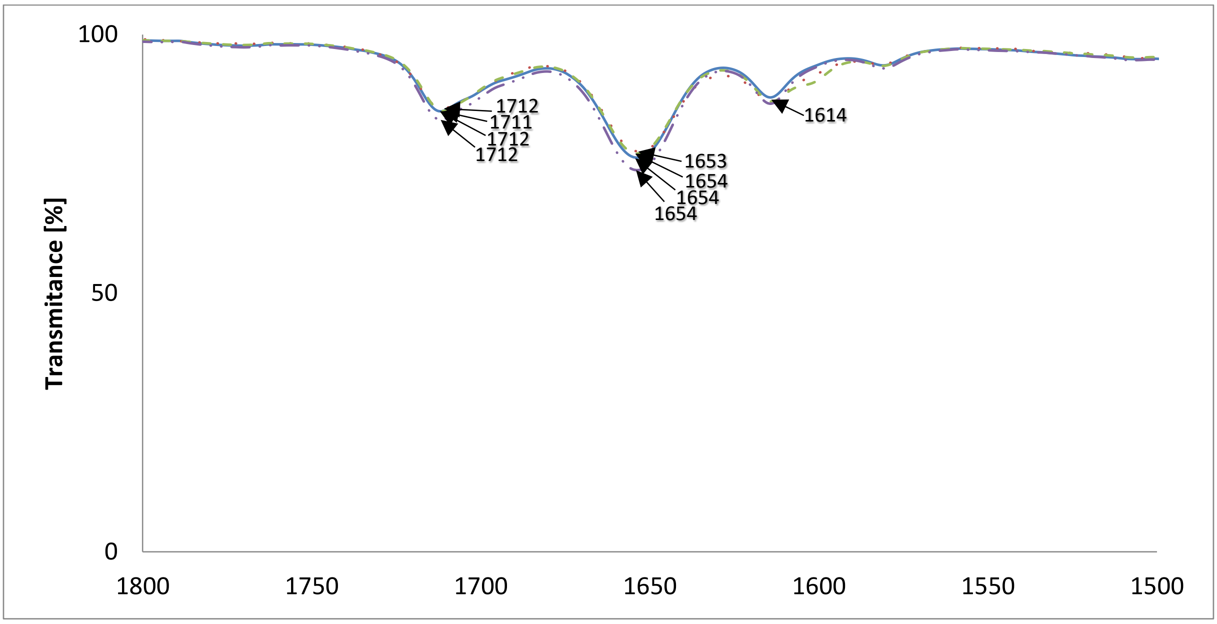

Fig. S8 The FT-IR spectra of (—) LasEico and its 1:1 complexes with cations: (– –) Li^+; (···) Na^+; (-··-) K^+; (a) 4000–400 cm^{-1}; (b) 1800–1500 cm^{-1}
